# Assessing Various Control Samples for Microarray Gene Expression Profiling of Laryngeal Squamous Cell Carcinoma

**DOI:** 10.3390/biom11040588

**Published:** 2021-04-16

**Authors:** Adam Ustaszewski, Magdalena Kostrzewska-Poczekaj, Joanna Janiszewska, Malgorzata Jarmuz-Szymczak, Malgorzata Wierzbicka, Joanna Marszal, Reidar Grénman, Maciej Giefing

**Affiliations:** 1 Institute of Human Genetics, Polish Academy of Sciences, Strzeszyńska 32, 60-479 Poznań, Poland; adam.ustaszewski@igcz.poznan.pl (A.U.); magdalena.kostrzewska-poczekaj@igcz.poznan.pl (M.K.-P.); joanna.janiszewska@igcz.poznan.pl (J.J.); malgorzata.jarmuz-szymczak@igcz.poznan.pl (M.J.-S.); otosk2@ump.edu.pl (M.W.); 2 Department of Oncology, Hematology and Bone Marrow Transplantation, Poznan University of Medical Sciences, 61-001 Poznań, Poland; 3 Department of Otolaryngology and Laryngological Oncology, Poznań University of Medical Sciences, 60-355 Poznań, Poland; joan.napierala@gmail.com; 4 Department of Otorhinolaryngology-Head and Neck Surgery, University of Turku and Turku University Hospital, 20520 Turku, Finland; seija.grenman@tyks.fi

**Keywords:** epithelial cells, gene expression profiling, laryngeal squamous cell carcinoma (LSCC), multidimensional scaling, primary tumor, microarray

## Abstract

Selection of optimal control samples is crucial in expression profiling tumor samples. To address this issue, we performed microarray expression profiling of control samples routinely used in head and neck squamous cell carcinoma studies: human bronchial and tracheal epithelial cells, squamous cells obtained by laser uvulopalatoplasty and tumor surgical margins. We compared the results using multidimensional scaling and hierarchical clustering versus tumor samples and laryngeal squamous cell carcinoma cell lines. A general observation from our study is that the analyzed cohorts separated according to two dominant factors: “malignancy”, which separated controls from malignant samples and “cell culture-microenvironment” which reflected the differences between cultured and non-cultured samples. In conclusion, we advocate the use of cultured epithelial cells as controls for gene expression profiling of cancer cell lines. In contrast, comparisons of gene expression profiles of cancer cell lines versus surgical margin controls should be treated with caution, whereas fresh frozen surgical margins seem to be appropriate for gene expression profiling of tumor samples.

## 1. Introduction

A major hurdle in the analysis of laryngeal squamous cell carcinoma (LSCC), is the proper selection of non-tumor controls for comparative analysis. This malignant neoplasm derives from the squamous epithelium of the upper aerodigestive tract, and therefore, cells of this origin are routinely used as controls in LSCC expression profiling. However, the biopsying of healthy individuals with the aim to obtain control tissues is unfeasible due to ethical reasons, hence, the collection of such tissues is limited to post mortem biopsying or surgical approaches. These include non-tumor oral and oropharyngeal epithelial tissues, obtained via uvulopalatopharyngoplasty (UPPP) of patients with obstructive sleep apnea [1,2,3], wisdom tooth extraction or frenectomy. Moreover, a widely used control source for LSCC expression studies includes tumor free surgical margins, obtained during the treatment of cancer patients [4,5,6,7].

Another type of control sample offered by several companies includes epithelial cell cultures obtained by bronchial brushings or by isolation of epithelial cells from cadaveric donations or non-tumor oral keratinocytes immortalized by transduction with retroviral vectors containing telomerase reverse transcriptase (hTERT) [8,9]. These cell lines derive from the oral epithelium, such as gingiva or buccal oral mucosa, which may be obtained during routine dental surgeries. Importantly, gene expression profiling of these tissues carries a bias due to long term cell culture in artificial conditions.

To address this issue, we used multidimensional scaling (MDS) [10] and hierarchical clustering [11]. MDS allows to detect latent variables from a previously obtained distance matrix, thereby revealing potential similarities and differences, which are not directly observed, of the particular dataset. Whereas, the basic idea of hierarchical clustering is to create specific groups (clusters) based on the similarity of the analyzed samples. The obtained clusters are further used to build a hierarchy which may be visualized as a dendrogram. By these methods, we compared four types of control samples (human bronchial epithelial cells, human tracheal epithelial cells, normal squamous cells and tumor surgical margins) being a frequent choice in LSCC gene expression profiling. These controls were compared versus primary tumor samples obtained from LSCC patients by surgical resection, as well as laryngeal squamous cell carcinoma (LSCC) cell lines. Our findings are potentially helpful in the selection of most suitable controls in gene expression profiling of head and neck tumors.

## 2. Materials and Methods

### 2.1. Control Samples Used for Gene Expression Profiling

- Human bronchial epithelial cells (HBEpiC, n = 5) and human tracheal epithelial cells (HTEpiC, n = 5), (frozen, cryopreserved cells at passage 0/1; ScienCell Research Laboratories, Carlsbad, CA, USA). The cells were cultured according to the protocol provided by ScienCell using the recommended Bronchial Epithelial Cell Medium (ScienCell Research Laboratories, Carlsbad, CA, USA) with 10% fetal bovine serum in T75 and T25 FLASK (Sarstedt Inc, Nümbrecht, Germany) coated with poly-L-lysine (PLL, ScienCell Research Laboratories, Carlsbad, CA, USA). The second passage, at confluence of approximately 70%, was used for expression profiling.
- Normal squamous cells were obtained from noncancerous patients using laser assisted uvulopalatoplasty (LAUP, n = 3). Cells were cultured prior to RNA isolation (approved by Ethical Review Board, no. 1156/18) in 25 cm^2^ flasks coated with 0.3 mg/mL PureCol collagen (Nutacon, Leimuiden, The Netherlands) in Dulbecco’s Modified Eagle Medium (Gibco, Thermo Fisher Scientific, Waltham, MA, USA) supplemented with 20% fetal bovine serum (Biochrom, Polgen, Łodz, Poland). The first passage of proliferating cells was used for expression profiling.
- Tumor surgical margins (n = 5) obtained during laryngectomy of LSCC patients treated at the Department of Otolaryngology at the University of Medical Sciences in Poznan, Poland. The specimens were fresh frozen and tissues lacking cancer cells according to histological examination were analyzed. The Institutional Ethical Review of the University of Medical Sciences approved tissue collection (no. 904/06), and informed consent was obtained from the patients.

### 2.2. Tumor Samples Used for Gene Expression Profiling

- LSCC tumor samples (n = 5) obtained during laryngectomy from patients treated at the Department of Otolaryngology University of Medical Sciences in Poznan, Poland. The specimens were characterized by pathological examination. Fresh frozen samples were used for RNA isolation. The Institutional Ethical Review of the University of Medical Sciences approved tissue collection (no. 904/06), and informed consent was obtained from the patients.
- Cultured in vitro six LSCC cell lines (n = 6) (UT-SCC-6A, UT-SCC-11, UT-SCC-19B, UT-SCC-29, UT-SCC-34 and UT-SCC-57 [12,13,14,15]) established at the University of Turku (Finland). The cells were grown in 25 cm^2^ flasks in Dulbecco’s Modified Eagle Medium (Gibco, Thermo Fisher Scientific, Waltham, MA, USA) supplemented with 10% fetal bovine serum (Biochrom, Polgen, Lodz, Poland). The gene expression profile of each LSCC cell line was performed twice (two separate microarray experiments) in order to obtain biological replicates.

### 2.3. RNA Isolation and Microarray Analysis

Total RNA was isolated using the Trizol reagent as described previously [16]. After removing the culture medium, the cells were immediately suspended in Trizol followed by cell lysis. Phase separation was performed by adding chloroform and RNA was precipitated by isopropanol and further resuspended in water with diethyl pyrocarbonate (DEPC). RNA integrity was measured using Agilent RNA 6000 Nano chip and Agilent 2100 Bioanalyzer, only samples with RIN > 7.2 were analyzed. Total RNA was shipped to ATLAS Biolabs (Berlin, Germany) to perform GeneChip Human Genome U133 Plus 2.0 Array (Affymetrix, Santa Clara, CA, USA) profiling. Microarray experiments were performed in three runs the resulting CEL files were normalized together using the MAS5 algorithm (mas5 function from affy R package) [17].

### 2.4. Bioinformatics

Global comparison of the expression profiles was performed using the MDS method with Manhattan distance calculation and hierarchical clustering using Ward’s method. The analysis was performed using the R packages base, stats, reshape2 [18,19] and the plots were prepared using the ggplot2 R package [20]. In order to establish the distances between the centroids of the analyzed cohorts, the Euclidean distances (Ed) were calculated. These values served as a numerical representation of the level of similarity among studied groups. For better clarity, all described Ed values are presented in simplified format (without scientific notation—e+06).

## 3. Results and Discussion

A general observation emerging from our study is that the analyzed cohorts separated according to two dominant factors that we called “malignancy”, which separated controls from malignant samples along dimension one and “cell culture”, which reflected the differences between cultured and non-cultured samples along dimension two. However, the separation along dimension two is probably also caused by the “microenvironment” that separated samples composed almost completely of epithelial cells (HBEpiC, HTEpiC) from samples with an admixture of other cell types (surgical margins, tumor samples and to some extent, LAUP).

**Figure 1:**
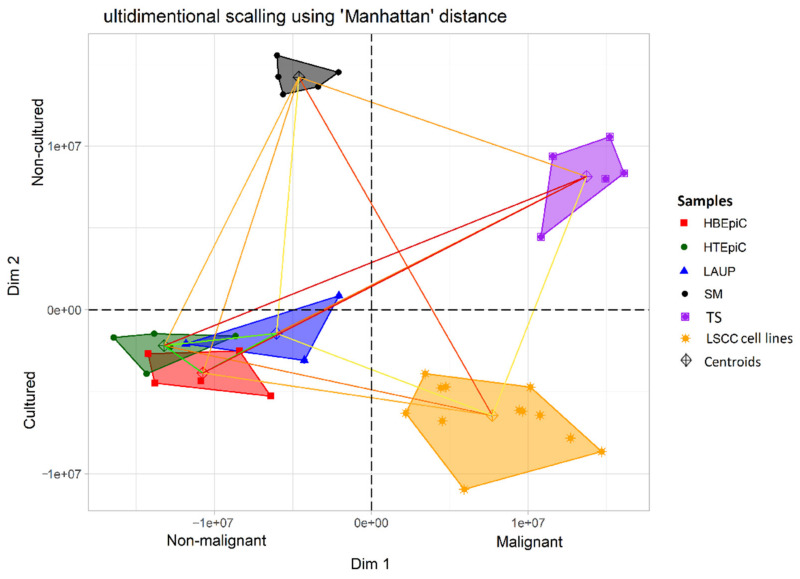
Visualization of MDS analysis using “Manhattan” method, five analyzed cohorts are shown: human bronchial epithelial cells (HBEpiC), human tracheal epithelial cells (HTEpiC), laser assisted uvulopalatoplasty (LAUP), tumor free surgical margins (SM), tumor samples (TS), and LSCC cell lines (LSCC cell lines) from two microarray experiments. The color of the lines represents the relative distance between cohorts.

**Figure 2:**
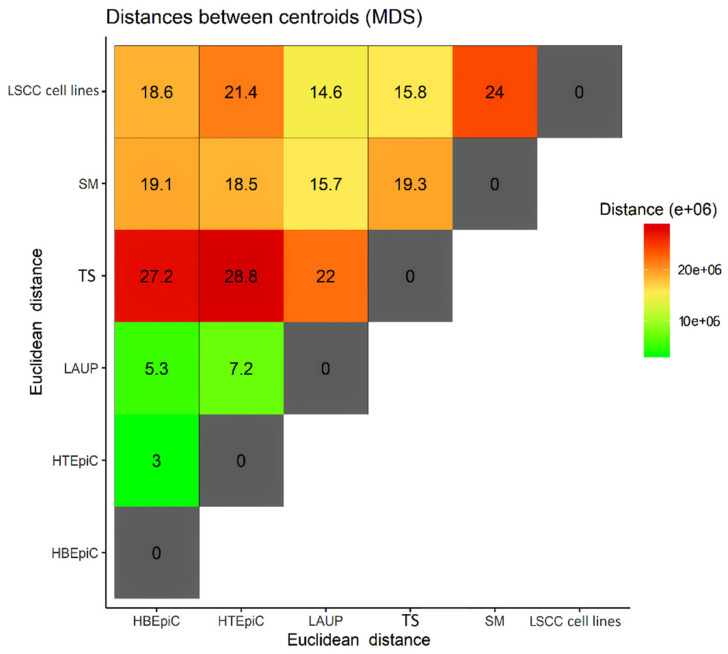
Distances between cohorts of studied samples.

**Figure 3:**
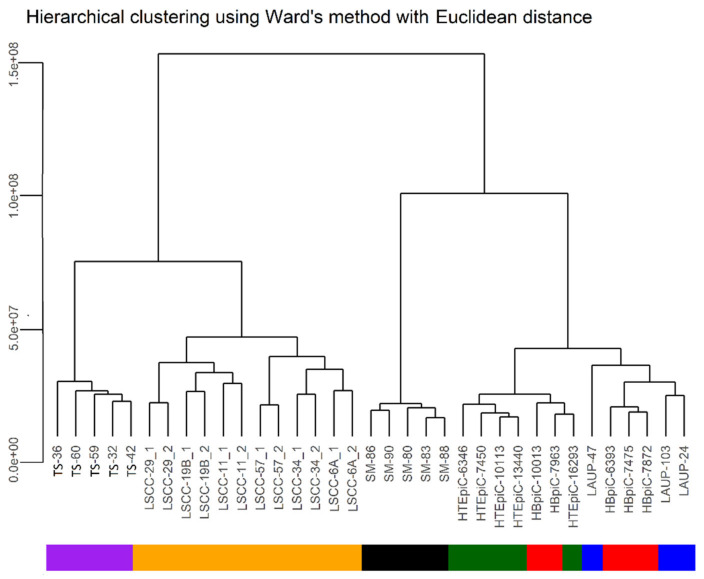
Hierarchical clustering of the studied cohorts using Ward’s method.

As expected, the gene expression profiles of the LSCC tumors significantly differed from all control groups and were presented as a distinct population in the MDS analysis (Ed range 14.6–24) (Figure 1, Figure 2 and Figure 3). Interestingly, LSCC cell lines and tumors were relatively distant (Ed 15.8), which further stresses the necessity to use adequate controls for each of these samples. 

An interesting finding of our study is the level of heterogeneity, observed among the LSCC cell lines and tumor samples. The level of heterogeneity within these samples exceeds the heterogeneity observed in the control epithelial cells (HBEpiC, HTEpiC) or surgical margins, respectively. This result underscores the known characteristic of high heterogeneity of LSCC tumors.

As expected, the bronchial and tracheal epithelial cells showed a high level of similarity in terms of gene expression profiles (Ed = 3). However, the LAUP samples only partially overlapped with the area occupied by HTEpiC and HBEpiC and, importantly, were located much closer to the surgical margins and tumor samples (Ed = 15.7; 22) than the HTEpiC and HBEpiC controls. This is probably caused by the admixture of fibroblasts and other non-epithelial cells in the LAUP samples. Importantly, there was a strong dissimilarity in gene expression profiles between cultured epithelial cells (HBEpiC, HTEpiC, LAUP) and surgical margins, indicating that the latter, although clearly different from the tumor samples and LSCC cell lines (Ed = 19.3; 24), are also a distinct entity from the other tested epithelial controls. Surgical margins were also closest to malignant samples along dimension one. This finding is interesting, especially in the sense that several authors reported that the apparently tumor cell free margins can harbor cancer cells and that epithelial cells in the margin can harbor epigenetic changes, predisposing them to tumor formation [21,22]. Additionally, the LAUP controls are shifted towards “malignancy” along dimension one which might reflect, for example, a smoking signature that mimics the neoplastic cells. 

Primary tumors, in turn, formed a group that was significantly separated from all other samples but clearly characterized by the shortest distance to LSCC cell lines (Ed = 15.8) and surgical margins (Ed = 19.3) indicating that features of both of these gene expression profiles may be found within this group. Moreover, the observed similarity in gene expression profiles of tumor samples and surgical margins along dimension two, in addition to the fact that both sample types were not cultured, might also reflect the influence of the microenvironment that HBEpiC, HTEpiC and cell lines lack.

Lastly, in order to determine the main biological processes and molecular functions differentiating surgical margins from cultured epithelial cells (HBEpiC, HTEpiC, LAUP), we performed a gene ontology analysis (GO). We used two freely available tools: The Gene Ontology Resource (Supplementary Materials, Table S1) [23,24] and DAVID (Supplementary Materials, Table S2) [25,26]. The input included 2239 differentially expressed genes (FC > 2.5, *p*-value < 0.01; 238 downregulated and 2001 upregulated in surgical margins) between the group of surgical margin samples and HBEpiC, HTEpiC, LAUP samples taken together (Supplementary Materials, Table S4). Among the enriched ontologies in the surgical margin samples, we identified several ontologies (FDR < 0.05; common for both tools) typical for tissues (Supplementary Materials, Table S3). These included angiogenesis (GO:0001525), immune response (GO:0006955) or response to wounding (GO:0009611) (Table 1). Therefore, the difference along dimension two reflects not only the influence of cell culturing, but also differences stemming from the complex nature of a tissue and the microenvironment of the surgical margin as compared to cell monolayer for the other controls.

## 4. Conclusions

Based on the presented results, we advocate the use of cultured HBEpiC and HTEpiC cells, and presumably also other types of cultured epithelial cells, as the ideal controls for gene expression profiling of LSCC cell lines. HBEpiC and HTEpiC controls are composed of a pure population of epithelial cells and as such, may be recognized as the normal counterparts of LSCC cells. Additionally, our data suggest that comparisons of gene expression profiles of cancer cell lines versus surgical margins should be treated with caution. Conversely, fresh frozen surgical margins and other controls obtained by surgical intervention seem to be appropriate for gene expression profiling of tumor samples as they allow one to eliminate the cell culturing bias. Moreover, the application of matched non-cancerous tissue seems more appropriate than a cohort of surgical margins collected from non-matched donors.

## Figures and Tables

**Table 1:** Gene ontologies enriched in the group of surgical margins compared to the HBEpiC, HTEpiC, LAUP samples and the involved differentially expressed genes (returned by both tools DAVID and Gene Ontology Resource).

GO Biological Process Name	Accession Number	Gene Ontology Resource (FDR)	DAVID (FDR)	Differentially Expressed Genes Involved in the Process (Common for DAVID and Gene Ontology Resource Tools)
Angiogenesis	GO:0001525	4.08 × 10^−14^	5.20 × 10^−13^	*ACVRL1, ANGPT1, ANGPT2, ANPEP, APOD, CALCRL, CCL2, CEACAM1, COL15A1, COL18A1, COL8A2, CXCL17, ECM1, ECSCR, ENPEP, EPHB2, ERAP1, FGF10, FGF18, FLT1, HEY1, HMOX1, HOXA3, HOXA7, HOXB3, JAM3, KDR, LEP, LEPR, MEOX2, MMP19, MMP2, NDNF, NOV, NRXN1, NRXN3, PDE3B, PIK3CG, PLXDC1, PLXND1, PRKD1, PTEN, PTPRB, RAMP2, ROBO4, RORA, S1PR1, SERPINE1, SHC1, SOX17, SOX18, TAL1, TEK, THSD7A, THY1, TIE1, TMEM100, TNFRSF12A, TNFSF12, VASH1, VAV3*
Immune response	GO:0006955	5.36 × 10^−50^	3.99 × 10^−58^	*ACKR4, ADAMDEC1, AIM2, C1QC, C1R, C3, C5AR1, C7, CCL13, CCL14, CCL18, CCL19, CCL2, CCL21, CCL22, CCL23, CCL26, CCL3, CCL4, CCL5, CCL8, CCR1, CCR2, CCR5, CCR6, CCR7, CD1A, CD1E, CD27, CD36, CD40LG, CD7, CD74, CD79B, CD86, CD8B, CD96, CFP, CLNK, CMKLR1, CRIP1, CST7, CTLA4, CTSG, CTSS, CTSW, CX3CL1, CXCL12, CXCL13, CXCL14, CXCL9, EDA, ENDOU, ENPP1, ENPP2, FCGR1A, FCGR1B, FCGR2B, FCGR2C, FCGR3A, FCGR3B, FYB, GBP2, GBP6, GEM, GPR183, GPR65, GZMA, HLA-B, HLA-C, HLA-DMA, HLA-DMB, HLA-DOA, HLA-DPA1, HLA-DPB1, HLA-DQA1, HLA-DQB1, HLA-DQB2, HLA-DRA, HLA-DRB1, HLA-DRB3, HLA-DRB4, HLA-DRB5, IGHA1, IGHA2, IGHD, IGHV1-69, IGHV3-13, IGHV3-23, IGHV3-30, IGHV3-33, IGHV3-48, IGHV3-53, IGKC, IGKV1-17, IGKV1-39, IGKV1-5, IGKV1D-39, IGKV2D-28, IGKV4-1, IGLC1, IGLV1-44, IGLV2-14, IGLV3-1, IGLV3-19, IGLV3-21, IGLV3-25, IGSF6, IL10, IL15, IL16, IL1A, IL1B, IL1R1, IL1R2, IL2RA, IL2RG, IL32, IL36A, IL7, IL7R, IRF8, JCHAIN, LAT, LCP2, LILRB2, LST1, LTB, LY75, MARCH1, MBP, MS4A2, NRROS, OAS1, OAS2, PKHD1L1, RGS1, SAMHD1, SEMA3C, SEMA4D, SMAD6, TENM1, TGFBR3, THBS1, TLR1, TLR10, TLR4, TNFRSF1B, TNFSF11, TNFSF12, TNFSF13B, TNFSF8, TRBC1, TRGC2, WAS, ZAP70*
Response to wounding	GO:0009611	6.86 × 10^−13^	5.06 × 10^−3^	*CCR1, CCR2, CX3CR1, DST, F2RL2, FGF7, GAP43, GRIN2A, HHEX, ITGB4, KLK6, LYVE1, PLLP, SLC1A2, SOX2, VASH1, VWF*

## Data Availability

The data presented in this study are available on request from the corresponding author.

## Supplementary Materials

The following are available online at https://www.mdpi.com/article/10.3390/biom11040588/s1, Table S1: GO Consortium PANTHER result, Table S2: DAVID result, Table S3: Common processes for both tools, Table S4: Differentially expressed genes, surgical margin samples vs. HTEpiC, HBpiC and LAUP.
